# Current and future antimicrobial treatment of gonorrhoea – the rapidly evolving *Neisseria gonorrhoeae* continues to challenge

**DOI:** 10.1186/s12879-015-1029-2

**Published:** 2015-08-21

**Authors:** Magnus Unemo

**Affiliations:** WHO Collaborating Centre for Gonorrhoea and Other STIs, National Reference Laboratory for Pathogenic Neisseria, Department of Laboratory Medicine, Microbiology, Faculty of Medicine and Health, Örebro University Hospital, Örebro University, SE-701 85 Örebro, Sweden

**Keywords:** Gonorrhoea, *Neisseria gonorrhoeae*, Treatment, Ceftriaxone, Azithromycin, Antimicrobial resistance, Treatment failure

## Abstract

*Neisseria gonorrhoeae* has developed antimicrobial resistance (AMR) to all drugs previously and currently recommended for empirical monotherapy of gonorrhoea. *In vitro* resistance, including high-level, to the last option ceftriaxone and sporadic failures to treat pharyngeal gonorrhoea with ceftriaxone have emerged. In response, empirical dual antimicrobial therapy (ceftriaxone 250–1000 mg plus azithromycin 1–2 g) has been introduced in several particularly high-income regions or countries. These treatment regimens appear currently effective and should be considered in all settings where local quality assured AMR data do not support other therapeutic options. However, the dual antimicrobial regimens, implemented in limited geographic regions, will not entirely prevent resistance emergence and, unfortunately, most likely it is only a matter of when, and not if, treatment failures with also these dual antimicrobial regimens will emerge. Accordingly, novel affordable antimicrobials for monotherapy or at least inclusion in new dual treatment regimens, which might need to be considered for all newly developed antimicrobials, are essential. Several of the recently developed antimicrobials deserve increased attention for potential future treatment of gonorrhoea. *In vitro* activity studies examining collections of geographically, temporally and genetically diverse gonococcal isolates, including multidrug-resistant strains particularly with resistance to ceftriaxone and azithromycin, are important. Furthermore, understanding of effects and biological fitness of current and emerging (*in vitro* induced/selected and *in vivo* emerged) genetic resistance mechanisms for these antimicrobials, prediction of resistance emergence, time-kill curve analysis to evaluate antibacterial activity, appropriate mice experiments, and correlates between genetic and phenotypic laboratory parameters, and clinical treatment outcomes, would also be valuable. Subsequently, appropriately designed, randomized controlled clinical trials evaluating efficacy, ideal dose, toxicity, adverse effects, cost, and pharmacokinetic/pharmacodynamics data for anogenital and, importantly, also pharyngeal gonorrhoea, i.e. because treatment failures initially emerge at this anatomical site. Finally, in the future treatment at first health care visit will ideally be individually-tailored, i.e. by novel rapid phenotypic AMR tests and/or genetic point of care AMR tests, including detection of gonococci, which will improve the management and public health control of gonorrhoea and AMR. Nevertheless, now is certainly the right time to readdress the challenges of developing a gonococcal vaccine.

## Review

### Introduction

The World Health Organization (WHO) estimated in 2008 that 106 million new gonorrhoea cases occur among adults annually worldwide [1]. If the gonococcal infections are not detected and/or appropriately treated, they can result in severe complications and sequelae such as pelvic inflammatory disease, infertility, ectopic pregnancy, first trimester abortion, neonatal conjunctivitis leading to blindness and, less frequently, male infertility and disseminated gonococcal infections. Gonorrhoea also increases the transmission and acquisition of HIV. Thus, gonorrhoea causes significant morbidity and socioeconomic consequences globally [1, 2]. In the absence of a gonococcal vaccine, public health control of gonorrhoea is relying on effective, accessible and affordable antimicrobial treatment, i.e., combined with appropriate prevention, diagnostics (index cases and traced sexual contacts), and epidemiological surveillance. The antimicrobial treatment should cure individual gonorrhoea cases, to reduce the risk of complications, and end further transmission of the infection, which is a crucial to decrease the gonorrhoea burden in a population.

Unfortunately, *Neisseria gonorrhoeae* has developed resistance to all antimicrobials introduced for treatment of gonorrhoea since the mid-1930s, when sulphonamides were introduced. The resistance to many antimicrobials has also rapidly, within only 1–2 decades, emerged and spread internationally [3–6]. The bacterium has utilized mainly all known mechanisms of antimicrobial resistance (AMR): inactivation of the antimicrobial, alteration of antimicrobial targets, increased export (e.g., through efflux pumps such as MtrCDE) and decreased uptake (e.g. through porins such as PorB). The mechanisms that change the permeability of the gonococcal cell are particularly concerning because these decrease the susceptibility to a wide range of antimicrobials with different modes of action, e.g., penicillins, cephalosporins, tetracyclines and macrolides [3, 5–8]. At present, the prevalence of *N. gonorrhoeae* resistance to most antimicrobials earlier recommended for treatment worldwide, such as sulphonamides, penicillins, earlier generation cephalosporins, tetracyclines, macrolides and fluoroquinolones, is high internationally [2–15]. In most countries, the only options for first-line empirical antimicrobial monotherapy are currently the extended-spectrum cephalosporins (ESCs) cefixime (oral) and particularly the more potent ceftriaxone (injectable) [2, 3, 5, 7, 8, 10–15].

### Conventional antimicrobial treatment of gonorrhoea

Treatment of gonorrhoea is mainly administered directly observed before any laboratory results are available, i.e., empirical therapy using first-line recommendations according to evidence-based management guidelines that are crucial to regularly update based on high quality surveillance data. Ideally, the recommended first-line therapy should be highly effective, widely available and affordable in appropriate quality and dose, lack toxicity, possible to administer as single dose, and cure >95 % of infected patients [2, 16]. However, levels of >1 % and >3 % AMR in high-frequency transmitting populations have also been suggested as thresholds for altering recommended treatment [16, 17]. Additional criteria, e.g. prevalence, local epidemiology, diagnostic tests, transmission frequency, sexual contact tracing strategies, and treatment strategies and cost, should ideally also be considered in this decision and the identical AMR threshold and recommended treatment regimen(s) may not be the most cost-effective solution in all settings and populations [3, 18, 19].

### Current antimicrobial treatment, ceftriaxone treatment failures, ceftriaxone resistant strains, and dual therapy

During the latest decade, cefixime 400 mg × 1 orally or ceftriaxone 125–1000 mg × 1 intramuscularly (IM) or intravenously (IV) has been recommended first-line for monotherapy of gonorrhoea in many countries globally [3–5, 7–9, 18, 20, 21]. However, since the first treatment failures with cefixime were verified in Japan in the early-2000s [22], failures have been verified in many countries worldwide, i.e. Norway, United Kingdom, Austria, France, Canada, and South Africa [23–29]. Most worryingly, sporadic treatment failures with ceftriaxone (250–1000 mg × 1), the last remaining option for empiric first-line monotherapy in many countries, have been verified in Japan, Australia, Sweden, and Slovenia [30–36]. The main characteristics of the verified treatment failures with ceftriaxone (n = 11) are described in Table 1.

**Table 1:** Characteristics of verified gonorrhoea treatment failures with ceftriaxone (250–1000 mg × 1) and causing gonococcal strain

Country, year	Ceftriaxone Therapy	Ceftriaxone MIC (mg/L)	*f*T_>MIC_, hours^a^	MLST/NG-MAST	Site of failure	Final successful treatment
Australia (n = 2), 2007 [31]	250 mg × 1	0.016-0.03 (Agar dilution)	41.4-50.3	ND/ST5, ST2740	Pharynx	Ceftriaxone 500 mg × 1/ Ceftriaxone 1 g × 1
Japan (n = 1), 2009 [30]	1 g × 1	4.0^b^ (Etest, XDR)	0	ST7363/ST4220	Pharynx	None^c^
Sweden (n = 1), 2010 [34]	250 mg × 1 and 500 mg × 1	0.125-0.25^b^ (Etest)	15.6-32.8	ST1901/ST2958	Pharynx	Ceftriaxone 1 g × 1
Australia (n = 1), 2010 [32]	500 mg × 1	0.03-0.06 (Agar dilution)	41.3-49.9	ND/ST1407, ST4950 (genogroup 1407)	Pharynx	Azithromycin 2 g × 1
Slovenia (n = 1), 2011 [36]	250 mg × 1	0.125^b^ (Etest)	24.3	ST1901/ST1407 (genogroup 1407)	Pharynx	Ceftriaxone 250 mg × 1 plus azithromycin 1 g × 1
Australia (n = 2), 2011 [33]	500 mg × 1	0.03-0.06 (Agar dilution)	41.3-49.9	ST1901/ST225, new variant of ST225	Pharynx	Ceftriaxone 1 g × 1 plus azithromycin 2 g × 1 or Ceftriaxone 1 g × 1
Sweden (n = 3), 2013–2014 [35]	500 mg × 1	0.064-0.125^b^ (Etest)	32.8-41.3	ST1901/ST3149, ST3149, ST4706 (genogroup 1407)	Pharynx	Ceftriaxone 1 g × 1

^a^Simulation of time of free ceftriaxone above MIC (*f *T_>MIC_) based on mean pharmacokinetic parameter values. Data from Chisholm et al. [52]

^b^Genetic cephalosporin resistance determinants (*penA*, *mtrR*, *penB*) elucidated [3, 5–8]

^c^The infection was considered to have resolved spontaneously within 3 months

*MIC* minimum inhibitory concentration, *MLST* multilocus sequence typing, *NG-MAST Neisseria gonorrhoeae* multi-antigen sequence typing, *ND* not determined, *ST* sequence type, *XDR* extensively drug-resistant [9]

Obviously, the number of verified treatment failures with ceftriaxone is low internationally. However, most likely these verified failures only represent the tip of the iceberg, because very few countries have active and quality assured surveillance and appropriately verify treatment failures. It is essential to strengthen this surveillance and follow-up of suspected and verified ceftriaxone treatment failures. WHO publications [2, 9, 16] recommend laboratory parameters to verify treatment failures, which ideally requires examining pre- and post-treatment isolates for ESC MICs, molecular epidemiological genotype, and genetic resistance determinants. Additionally, a detailed clinical history that excludes reinfection and records the treatment regimen(s) used is mandatory.

Briefly, the ceftriaxone MICs of the gonococcal isolates causing the ceftriaxone treatment failures ranged from 0.016 to 4 mg/L. Seven (88 %) of the eight isolates genotyped with multilocus sequence typing (MLST) were assigned to ST1901. Six (55 %) failures were caused by gonococcal strains belonging to the *N. gonorrhoeae* multiantigen sequence typing (NG-MAST) ST1407 or genetically closely related NG-MAST STs, such as ST2958, ST3149, ST4706, and ST4950, of which five (45 %) belong to NG-MAST genogroup 1407 [37]. However, the failure to treat pharyngeal gonorrhoea in a female commercial sex worker with ceftriaxone 1 g × 1 in Kyoto, Japan, was caused by a strain assigned as MLST ST7363 and NG-MAST ST4220 (Table 1). This strain was the first verified extensively drug-resistant (XDR [9]) *N. gonorrhoeae* strain (‘H041’; the first gonococcal ‘superbug’), which displayed high-level resistance to ceftriaxone (MIC = 2-4 mg/L) [30]. Only two years later (2011), two additional superbugs were identified in men-who-have-sex-with-men (MSM) in France [26] and Spain [38], which are suspected to belong to the identical strain (‘F89’) and may represent the first international transmission of a high-level ceftriaxone resistant gonococcal strain. In 2014, a ceftriaxone resistant strain with genetic similarities to H041 was reported in Australia [39]. However, this strain had a lower ceftriaxone MIC compared to H041 and F89 (MIC: 0.5 mg/L versus 2–4 mg/L using Etest), and sporadic gonococcal strains with this low-level ceftriaxone resistance have been previously described internationally [25, 40, 41]. The main characteristics of the verified superbugs and examples of sporadic gonococcal strains with ceftriaxone MIC = 0.5 mg/L are described in Table 2.

**Table 2:** Main characteristics of the verified *Neisseria gonorrhoeae* superbugs and examples of sporadic gonococcal strains with ceftriaxone MIC = 0.5 mg/L

Country, year	Ceftriaxone MIC (mg/L)	*f*T_>MIC_ (hours) with ceftriaxone 250 mg × 1 (1 g × 1)^a^	MLST	NG-MAST	PBP2 sequence variant [30]
Japan, 2009 “H041” [30]	4 (Etest)	0-0 (0–5.6)	ST7363	ST4220	C (X + 12 amino acid alterations; new key resistance alterations: A311V, T316P, T483S [45]) ^b^
France, 2011 “F89” [26]	2 (Etest)	0-0 (0–20.3)	ST1901	ST1407	CI (XXXIV + A501P)^b^
Spain, 2011 “F89” [38]^c^	2 (Etest)	0-0 (0–20.3)	ST1901	ST1407	CI (XXXIV + A501P)^b^
Japan, 2000–2001 [40]	0.5 (Agar dilution)	0-19.8 (11.1-49.8)	ND	ND	X-variant (X + N575Δ + V576A)^b^
China, 2007 [41]	0.5 (Agar dilution)	0-19.8 (11.1-49.8)	ND	ST2288	XVII
Austria, 2011 [25]	0.5 (Etest)	0-19.8 (11.1-49.8)	ST1901	ST1407	XXXIV + T534A^b^
Australia, 2014 “A8806” [39]	0.5 (Agar dilution)	0-19.8 (11.1-49.8)	ST7363	ST4015^d^	C-variant (including two of the three key alterations in H041: A311V and T483S)^b^

^a^Monte Carlo simulation, taking into account diversity inherent within patient populations, showing 95 % confidence intervals of time (h) of free ceftriaxone above MIC (*f *T_>MIC_). Data from Chisholm et al. [52]

^b^Mosaic PBP2 sequence variant [30]

^c^Possibly identical to the earlier identified French superbug [26] and represented the first international transmission of a high-level ceftriaxone resistant gonococcal strain

^d^Compared to the superbug H041 [30], identical *tbpB* allele (10) and a *porB* allele (1059) that only differed by 6 %

*MIC* minimum inhibitory concentration, *MLST* multilocus sequence typing, *NG-MAST Neisseria gonorrhoeae* multi-antigen sequence typing, *PBP2* penicillin-binding protein 2, *ND* not determined, *ST* sequence type

Briefly, the first verified gonococcal superbug H041 had a ceftriaxone MIC of 4 mg/L using Etest and was assigned to NG-MAST ST4220 and MLST ST7363 [30], an MLST clone that has been prevalent and caused many of the early cefixime treatment failures in Japan. The gonococcal strains causing these early cefixime treatment failures had a mosaic penicillin-binding protein 2 (PBP2) X sequence variant [3, 8, 30, 42–44]. However, H041 had developed also high-level ceftriaxone resistance by 12 additional amino acid alterations in PBP2 X [30], of which the novel key resistance amino acid alterations were A311V, T316P, T483S [45]. The A8806 strain recently detected in Australia (ceftriaxone MIC = 0.5 mg/L) showed some key genetic similarities to H041, including the identical MLST ST7363, similar NG-MAST ST, and shared two (A311V and T483S) of the three PBP2 alterations pivotal to the high-level ceftriaxone resistance [39, 45]. Noteworthy, three of the five additional isolates with ceftriaxone MIC ≥ 0.5 mg/L were assigned as MLST ST1901 and NG-MAST ST1407 (Table 2). This clone has been traced back to 2003 in Japan, accounting for most of the decreased susceptibility and resistance to ESCs in Europe, and basically spread globally [3, 8, 23–27, 29, 32, 35–38, 43, 44, 46, 47]. Noteworthy, although ST1407 has been the most prevalent NG-MAST ST of MLST ST1901 in Europe, many NG-MAST STs of this MLST clone have been identified globally, particularly in Japan, where ST1901 replaced ST7363 as the most prevalent MLST clone already in the early 2000s [3, 8, 43, 44]. Most frequently, this clone has had a mosaic PBP2 XXXIV [3, 8, 23, 27, 35, 36], however, in all these three isolates the PBP2 had mutated and included one additional mutation, i.e., A501P (French and Spanish strain) or T534A (Swedish strain) [25, 26, 38]. Undoubtedly, the superbugs and these additional sporadic strains illustrate that gonococci have different ways to develop ceftriaxone, including high-level, resistance and that only one or a few mutations in PBP2 are required for ceftriaxone resistance in a large proportion of strains circulating worldwide [3, 8, 14, 23–27, 29, 30, 32, 35–40, 42–44, 46–49]. Several additional ceftriaxone resistant strains may already be circulating but are undetected due to the suboptimal AMR surveillance in many settings internationally. Most noteworthy, the gonococcal strain detected in China in 2007 (ceftriaxone MIC = 0.5 mg/L; non-mosaic PBP2 XVII) emphasizes that gonococci can also develop ceftriaxone resistance without a mosaic PBP2 [41]. In the non-mosaic PBP2 XVII, the A501V and G542S mutations are suspected to be involved in the ceftriaxone resistance, i.e. most likely together with the resistance determinants *mtrR* and *penB* [3, 8, 41, 45, 50, 51]. Notably, particularly in Asia many strains with a ceftriaxone MIC = 0.25 mg/L, i.e. ceftriaxone resistant according to the European resistance breakpoints (www.eucast.org), which lack a mosaic PBP2 are also circulating. E.g., gonococcal strains with ceftriaxone MIC = 0.25 mg/L and non-mosaic PBP2s have been described in China (PBP2 XIII with A501TV and P551S [41]), South Korea (PBP2 IV and V with G542S [48], and XIII with A501TV and P551S [49]), and Vietnam (PBP2 XVIII with A501T and G542S [51]).

Regarding pharmacodynamics, it has been suggested that a time of free ESC above MIC (*f*T_>MIC_) of 20–24 hours is required for treatment with ESCs [52]. Applying these figures on the gonococcal superbugs and other sporadic strains with ceftriaxone MICs ≥ 0.5 mg/L, according to Monte Carlo simulations sufficient *f*T_>MIC_ is not reached for any strain even at upper 95 % confidence interval (CI) when using ceftriaxone 250 mg × 1. Furthermore, even with ceftriaxone 1 g × 1, 20–24 hours of *f*T_>MIC_ will be reached in only very few, if any, patients infected with the superbugs and additionally it will not be reached in many of the patients infected even with the strains showing ceftriaxone MIC = 0.5 mg/L (Table 2). However, several of the ceftriaxone treatment failures have been caused by ceftriaxone susceptible gonococcal strains with a relatively low ceftriaxone MIC (0.016-0.125 mg/L), and in many of these cases the *f*T_>MIC_ should have been substantially longer than 20–24 hours (Table 1). These treatment failures were all for pharyngeal gonorrhoea and, most likely, reflect the difficulties in treating pharyngeal gonorrhoea compared with urogenital gonorrhoea [3, 8, 9, 13, 30–36, 53–55]. Sufficient understanding regarding the complex process when antimicrobials penetrate into the pharyngeal mucosa, where also the presence of inflammation and pharmacokinetic properties of the antimicrobial are important factors, is lacking. It is crucial to elucidate why many antimicrobials, at least in some patients, appear to achieve suboptimal concentrations in tonsillar and other oropharyngeal tissues [55]. Appropriate pharmacokinetic/pharmacodynamic studies and/or optimized simulations with currently and futurely used antimicrobials are essential for gonorrhoea, particularly pharyngeal infection. It has also been suggested that ESC resistance initially emerged in commensal *Neisseria* spp., which act as a reservoir of AMR genes that are easily transferred to gonococci through transformation, particularly in pharyngeal gonorrhoea [3, 7–9, 42, 55–57]. Pharyngeal gonorrhoea is mostly asymptomatic, and gonococci and commensal *Neisseria* spp. can coexist for long time periods in the pharynx and share AMR genes and other genetic material. Accordingly, an enhanced focus on early detection (screening of high-risk populations, such as MSM, with nucleic acid amplification tests (NAATs) should be considered) and appropriate treatment of pharyngeal gonorrhoea is imperative [2,3,8,13,56,].

The emergence of ceftriaxone treatment failures and particularly the superbugs with high-level ceftriaxone resistance [26, 30, 38], combined with resistance to mainly all other gonorrhoea antimicrobials, resulted in a fear that gonorrhoea might become exceedingly-difficult-to-treat or even untreatable. Consequently, the WHO published the ‘Global Action Plan to Control the Spread and Impact of Antimicrobial Resistance in *Neisseria gonorrhoeae*’ [2, 58], and the European Centre for Disease Prevention and Control (ECDC) [59] and the US Centers for Disease Control and Prevention (CDC) published region-specific response plans [60]. In general, all these plans request more holistic actions, i.e., to improve early prevention, diagnosis, contact tracing, treatment, including test-of-cure, and epidemiological surveillance of gonorrhoea cases. It was also stated essential to, nationally and internationally, significantly enhance the surveillance of AMR (maintaining culture is imperative), treatment failures and antimicrobial use/misuse locally (strong antimicrobial stewardship crucial). Evidently, gonococcal AMR data were lacking in many settings globally and, accordingly, the WHO Global Gonococcal Antimicrobial Surveillance Programme (WHO Global GASP) was reinitiated in 2009, in close liaison with other AMR surveillance initiatives, to enable a coordinated global response [58]. During recent years, dual antimicrobial therapy (mainly ceftriaxone 250–500 mg × 1 and azithromycin 1–2 g × 1) for empirical gonorrhoea treatment has also been introduced in Europe, Australia, USA, Canada, and some additional countries (Table 3).

**Table 3:** Recommended and alternative treatments for uncomplicated *Neisseria gonorrhoeae* infections of the urethra, cervix, rectum and pharynx in adults and youth in Europe, United Kingdom, Germany, Australia, USA, and Canada

	Europe [61]	United Kingdom [62]	Germany [63]	Australia [64]	USA [65]	Canada [66]
*Recommended (first-line) regimens for anogenital infections* ^*a*^	Ceftriaxone 500 mg × 1 IM	Ceftriaxone 500 mg × 1 IM	Ceftriaxone 1 g × 1 IM/IV	Ceftriaxone 500 mg × 1 IM	Ceftriaxone 250 mg × 1 IM	Ceftriaxone 250 mg × 1 IM
	PLUS	PLUS	PLUS	PLUS	PLUS	PLUS
	Azithromycin 2 g × 1 orally^*b*^	Azithromycin 1 g × 1 orally	Azithromycin 1.5 g × 1 orally	Azithromycin 1 g × 1 orally	Azithromycin 1 g × 1 orally	Azithromycin 1 g × 1 orally
						OR
						Cefixime 800 mg × 1 orally
						PLUS
						Azithromycin 1 g × 1 orally
*Alternative regimens for anogenital infections* ^*a*^	1. Cefixime 400 mg × 1 orally	All the options below should be taken with Azithromycin 1 g × 1 orally.^*c*^	If IM/IV injection is not possible:	Alternative treatments are not recommended because of high levels of resistance, except for some remote Australian locations and severe allergic reactions.	If ceftriaxone is not available:	Spectinomycin 2 g × 1 IM
	PLUS	→ Cefixime 400 mg × 1 orally. Only if an injection contra-indicated or refused.	Cefixime 800 mg × 1 orally		Cefixime 400 mg × 1 orally	PLUS
	Azithromycin 2 g × 1 orally.	→ Spectinomycin 2 g × 1 IM.	PLUS		PLUS	Azithromycin 1 g × 1 orally
	Only if ceftriaxone not available or administration of injectable antimicrobials not possible or refused.	→ Cefotaxime 500 mg × 1 IM or Cefoxitin 2 g × 1 IM PLUS probenecid 1 g × 1 orally.	Azithromycin 1.5 g × 1 orally		Azithromycin 1 g × 1 orally	OR
	2. Ceftriaxone 500 mg × 1 IM.	Other cephalosporins offer no advantage in terms of efficacy and pharmacokinetics over ceftriaxone or cefixime.	or if *N. gonorrhoeae* known to be susceptible:			Azithromycin 2 g × 1 orally
	Only if azithromycin not available or patient unable to take oral medication.^*c*^	→ Cefpodoxime with caution at a dose of 400 mg × 1 orally.	→ Cefixime 400 mg × 1 orally			
	3. Spectinomycin 2 g × 1 IM	→ When an infection is known before treatment to be quinolone susceptible, ciprofloxacin 500 mg × 1 orally or ofloxacin 400 mg × 1 orally.	→ Ciprofloxacin 500 mg × 1 orally or Ofloxacin 400 mg × 1 orally.			
	PLUS		→ Azithromycin 1.5 g × 1 orally			
	Azithromycin 2 g × 1 orally.					
	E.g., if resistance to extended-spectrum cephalosporins is identified or suspected, or patient has history of penicillin anaphylaxis or cephalosporin allergy.					
*Recommended treatment for pharyngeal infections*	Identical regimen as recommended for anogenital infections.	Identical regimen as recommended for anogenital infections.	Identical regimen as recommended for anogenital infections.	Identical regimen as recommended for anogenital infections.	Identical regimen as recommended for anogenital infections.	Ceftriaxone 250 mg × 1 IM
		OR if *N. gonorrhoeae* known to be quinolone susceptible:	OR if *N. gonorrhoeae* known to be susceptible:			PLUS
		→ Ciprofloxacin 500 mg × 1 orally or Ofloxacin 400 mg × 1 orally.	→ Ciprofloxacin 500 mg × 1 orally or Ofloxacin 400 mg × 1 orally.			Azithromycin 1 g × 1 orally
			→ Azithromycin 1.5 g × 1 orally			*Alternatives:*
						Cefixime 800 mg × 1 orally
						PLUS
						Azithromycin 1 g × 1 orally
						OR
						Azithromycin 2 g × 1 orally.
*Recommended regimen when extended-spectrum cephalosporin resistance identified or failure with recommended dual regimen*	→ Ceftriaxone 1 g × 1 IM	No recommendation.	No recommendation.	No recommendation.	→ Retreatment with recommended dual regimen.	It is strongly recommended that treatment be guided by antimicrobial susceptibility test results to determine the appropriate antimicrobial agent in consultation with an expert in infectious diseases and local public health authorities.
	PLUS				→ Gemifloxacin 320 mg × 1 orally	
					PLUS	
					Azithromycin 2 g × 1	
	Azithromycin 2 g × 1 orally.				OR	
	→ Gentamicin 240 mg × 1 IM				Gentamicin 240 mg × 1 IM	
	PLUS				PLUS	
	Azithromycin 2 g × 1 orally.^*b*^				Azithromycin 2 g × 1 can be considered.	

*IM* intramuscularly, *IV* intravenously

^*a*^Uncomplicated gonococcal infections of the cervix, urethra and rectum

^*b*^Azithromycin tablets may be taken with or without food but gastrointestinal side effects can be less if taken after food

^*c*^Co-infection with *Chlamydia trachomatis* is common in young (<30 years) heterosexual individuals and men who have sex with men (MSM) with gonorrhoea. If treatment for gonorrhoea does not include azithromycin, treatment with azithromycin 1 g × 1 orally or doxycycline 100 mg orally twice daily for 7 days should be given for possible chlamydial co-infection unless co-infection has been excluded with nucleic acid amplification test (NAAT)

Briefly, all regions or countries, with exception of Canada, recommend only ceftriaxone plus azithromycin as first-line [61–66]. However, the recommended doses of ceftriaxone vary, i.e. range from 250 mg × 1 (USA and Canada) to 1 g × 1 (Germany), and the doses of azithromycin range from 1 g × 1 (USA, Canada, UK and Australia) to 2 g × 1 (Europe) (Table 3). Appropriate clinical data to support the different recommended doses of ceftriaxone and azithromycin (in the combination therapy) for the currently circulating gonococcal population are mainly lacking. Instead, these treatment regimens were based on early clinical efficacy trials [3, 7, 54, 67–72], pharmacokinetic/pharmacodynamic simulations [52], *in vitro* AMR surveillance data, anticipated trends in AMR, case reports of treatment failures [22–26, 30, 31, 34, 36, 73], and expert consultations. No other currently available and evaluated injectable cephalosporin (e.g., ceftizoxime, cefoxitin with probenecid, and cefotaxime) offers any advantages over ceftriaxone in terms of efficacy and pharmokinetics/pharmacodynamics, and efficacy for pharyngeal infection is less certain [3, 8, 9, 21, 61, 65, 67–72, 74]. In Canada, also an oral first-line therapy is recommended, i.e. cefixime 800 mg × 1 plus azithromycin 1 g × 1. Mainly early evidence indicated that cefixime 800 mg × 1 was safe and effective in treating gonorrhoea [66, 69, 71, 72, 75, 76]. Pharmacodynamic studies and/or simulations have also shown that, compared to 400 mg × 1, 800 mg of cefixime (particularly administered as 400 mg × 2, 6 hours apart) substantially increases the *f*T_>MIC_ of cefixime [22, 52]. However, in most countries cefixime is only licensed for the currently or previously used 400 mg × 1, due to the more frequent gastrointestinal adverse effects observed with 800 mg × 1 [70], and treatment failures with also cefixime 800 mg × 1 have been verified [28].

Two different novel dual antimicrobial regimens have also been evaluated for treatment of uncomplicated urogenital gonorrhoea, i.e., gentamicin (240 mg × 1 IM) plus azithromycin (2 g × 1 orally), and gemifloxacin (320 mg × 1 orally) plus azithromycin (2 g × 1 orally) [77]. The cure rate was 100 % with gentamicin + azithromycin and 99.5 % with gemifloxacin + azithromycin, but gastrointestinal adverse effects were frequent. E.g., 3.3 % and 7.7 % of patients, respectively, vomited within one hour of treatment, which necessitated retreatment with ceftriaxone and azithromycin [77]. Nevertheless, these two therapeutic regimens can be considered in the presence of ceftriaxone resistance, treatment failure with recommended regimen, or ESC allergy.

### Future treatment of gonorrhoea

Future treatment should be in strict concordance with continuously updated evidence-based management guidelines, informed by quality assured surveillance of local AMR and also treatment failures. Dual antimicrobial therapy (ceftriaxone and azithromycin [61–66]), which also eradicates concurrent chlamydial infections and many concurrent *Mycoplasma genitalium* infections, should be considered in all settings where local quality assured AMR data do not support other therapeutic options. Despite that the dual antimicrobial regimens with ceftriaxone and azithromycin may not entirely prevent resistance emergence [3, 8, 78], they will mitigate the spread of resistant strains. Nevertheless, after strict evaluation (effectiveness and compliance) multiple doses of single antimicrobials should also be considered. An oral treatment regimen (single or dual antimicrobials) would be exceedingly valuable and also allow patient-delivered partner therapy that at least in some settings may decrease the gonorrhoea prevalence at population level [79, 80].

Ideally, treatment at first health care visit will also be individually-tailored, i.e. by novel rapid phenotypic AMR tests, e.g. broth microdilution MIC assays, or genetic point of care (POC) AMR tests, including detection of gonococci. This will ensure a rational antimicrobial use (including sparing last-line antimicrobials), timely notification of sexual contacts, slow the AMR development, and improve the public health control of both gonorrhoea and AMR [3, 4, 6, 81, 82]. No commercially available gonococcal NAAT detects any AMR determinants. However, laboratory-developed NAATs have been designed and used for identification of genetic AMR determinants involved in resistance to penicillins, tetracyclines, macrolides, fluoroquinolones, cephalosporins, and multidrug-resistance [3–7, 83–87]. Some “strain-specific” NAATs detecting the key ESC resistance mutations in the superbugs H041 [30] and F89 [26, 38] have also been developed [88, 89]. However, genetic AMR testing will not entirely replace phenotypic AMR testing because the relationships between phenotypes and genotypes are not ideal, genetic methods can only identify known AMR determinants, the sensitivity and/or specificity in the prediction of AMR or antimicrobial susceptibility is suboptimal (particularly for ESCs with their ongoing resistance evolution involving many different genes, mutations, and their epistasis), and new AMR determinants continuously evolve [3–5, 8, 14]. Tests requiring continual updating with new targets will not be profitable for commercial companies manufacturing NAATs. In addition, several of the gonococcal AMR determinants, e.g. mosaic *penA* alleles, originate in commensal *Neisseria* species, which makes it difficult to predict gonococcal AMR in pharyngeal samples [3, 8, 9]. Further research is crucial to continuously identify new AMR determinants and appropriately evaluate how current and future molecular AMR assays can supplement phenotypic AMR surveillance and ultimately guide individually-tailored treatment [3, 4, 6, 8, 14]. At present, at least for AMR surveillance ciprofloxacin susceptibility is relatively easy to predict, azithromycin susceptibility or resistance can be indicated, and decreased susceptibility or resistance to ESCs can be predicted, although with a low specificity, by detecting mosaic *penA* alleles. Nevertheless, also non-mosaic PBP2 sequences can cause ceftriaxone resistance [41, 48, 49, 51]. High-throughput genome sequencing [46, 47, 90–92], transcriptomics and other novel technologies will likely revolutionize the genetic AMR prediction and molecular epidemiological investigations of both gonococcal isolates and gonococcal NAAT positive samples.

### Future treatment options for gonorrhoea

The current dual antimicrobial treatment regimens (ceftriaxone plus azithromycin [61–66]) appear to be effective. However, the susceptibility to ceftriaxone in gonococci has decreased globally, azithromycin resistance is relatively prevalent in many countries, concomitant resistance to ceftriaxone and azithromycin has been identified in several countries, and the dual antimicrobial regimens are not affordable in many less-resourced settings [3, 8, 14, 15, 18, 78]. Furthermore, treatment failures with even azithromycin 2 g × 1 have been verified [93–95] and gonococcal strains with high-level resistance to azithromycin (MIC ≥ 256 mg/L) have been described in Scotland [96], United Kingdom [97], Ireland [98], Italy [99], Sweden [100], USA [101], Argentina [102], and Australia [103]. Accordingly, no treatment failure with dual antimicrobial therapy (ceftriaxone 250–500 mg × 1 plus azithromycin 1–2 g × 1) has been verified yet, nevertheless, most likely it is only a matter of when, and not if, treatment failures with these dual antimicrobial regimens will emerge. Consequently, novel affordable antimicrobials for monotherapy or at least inclusion in new dual treatment regimens for gonorrhoea, which might need to be considered for all newly designed antimicrobials, are essential.

The earlier frequently used aminocyclitol spectinomycin (2 g × 1 IM) is effective for treatment of anogenital gonorrhoea, however, the efficacy against pharyngeal infection is low (51.8 %; 95 %CI: 38.7 %-64.9 %) [53] and it is currently not available in many countries [3, 61, 62, 65]. However, the *in vitro* susceptibility to spectinomycin is exceedingly high worldwide, including in South Korea where it has been very frequently used for treatment [3, 5, 7, 8, 18, 49, 51, 61, 104–109]. Accordingly, in South Korea 53-58 % of gonorrhoea patients in 2002–2006 [109] and 52-73 % in 2009–2012 were treated with spectinomycin [49]. Despite this exceedingly high spectinomycin usage, spectinomycin resistance has not been reported since 1993 in South Korea [49]. Thus, the spread of spectinomycin resistance in the 1980s [110–112] may reflect more uncontrolled usage of spectinomycin and the transmission of some few successful spectinomycin resistant gonococcal strains. Research regarding biological fitness cost of spectinomycin resistance would be valuable, and in fact spectinomycin might be underestimated for treatment of gonorrhoea. This is particularly in dual antimicrobial therapy together with azithromycin 1–2 g × 1, which are alternative therapeutic regimens recommended in the European [61] and Canadian [66] gonorrhoea management guidelines, that will also cover pharyngeal gonorrhoea and potentially mitigate emergence of resistance to both spectinmycin and azithromycin.

Other “old” antimicrobials that have been suggested for future empirical monotherapy of gonorrhoea include the injectable carbapenem ertapenem [113, 114], oral fosfomycin [115], and injectable aminoglycoside gentamicin, which has been used as first-line treatment, 240 mg × 1 IM together with doxycycline in syndromic management, in Malawi since 1993 without any reported emergence of *in vitro* resistance [3, 7, 61, 65, 67, 77, 116–119]. However, disadvantages with these antimicrobials include that *in vitro* resistance is rapidly selected (fosfomycin) or decreased susceptibility already exist (ertapenem [113, 114]), evidence-based correlates between MICs, pharmacokinetic/pharmacodynamic parameters and gonorrhoea treatment outcome are lacking (gentamicin, fosfomycin and ertapenem), and mainly no recent clinical data exist for empiric monotherapy of urogenital and particularly extragenital gonorrhoea (gentamicin, fosfomycin and ertapenem). Consequently, these antimicrobials are most likely mainly options for ceftriaxone-resistant gonorrhoea, ESC allergy and/or in noval dual antimicrobial treatment regimens. Nevertheless, some small observational or controlled studies mainly from the 1970s and 1980s evaluated gentamicin for monotherapy of gonorrhoea. Two recent meta-analyses of several of these studies reported that a single dose of gentamicin resulted in cure rates of only 62-98 % [119] and a pooled cure rate of 91.5 % (95 %CI: 88-94 %) [118]. However, these early gentamicin studies were mainly small, of low quality and in general provided insufficient data. Consequently, a multi-centre (n = 8), parallel group, investigator-blinded, non-inferiority, randomized, controlled Phase 3 clinical trial has been recently initiated. This study aims to recruit 720 patients with uncomplicated urogenital, pharyngeal and rectal gonorrhoea. Treatment with gentamicin 240 mg × 1 IM (n = 360) compared to ceftriaxone 500 mg × 1 IM (n = 360), plus azithromycin 1 g × 1 orally to each arm, will be evaluated, in regard to clinical effectiveness, cost-effectiveness and safety (www.research.uhb.nhs.uk/gtog).

Many derivates of earlier used antimicrobials have also been evaluated *in vitro* against gonococcal strains recent years. For example, several new fluoroquinolones, e.g. avarofloxacin (JNJ-Q2), sitafloxacin, WQ-3810, and delafloxacin, have shown relatively high potency against gonococci, including ciprofloxacin-resistant isolates [120–123]. The fluorocycline eravacycline (TP-434) and glycylcycline tigecycline (family: tetracyclines) also appear to be effective against gonococci [124, 125]. Nevertheless, a small fraction of administered tigecycline is excreted unchanged in urine, which might question the use in gonorrhoea treatment [126–128]. The lipoglycopeptide dalbavancin and two new 2-acyl carbapenems (SM-295291 and SM-369926) have shown a high activity against a limited number of gonococcal isolates [129, 130]. Finally, the two “bicyclic macrolides” modithromycin (EDP-420) and EDP-322 displayed relatively high activity against azithromycin-resistant, ESC-resistant and multidrug-resistant (MDR) gonococci, but high-level azithromycin resistant gonococcal isolates (MIC ≥ 256 mg/L) were resistant also to modithromycin and EDP-322 [131]. Unfortunately, no clinical efficacy data for treatment of gonorrhoea exist for any of these antimicrobials. More advanced in the development is the novel oral fluoroketolide solithromycin (family: macrolides) that has proved to have a high activity against gonococci, including azithromycin-resistant, ESC-resistant and MDR isolates [132]. Solithromycin has three binding sites on the bacterial ribosome (compared with two for other macrolides), which likely result in a higher antibacterial activity and delay resistance emergence [133]. However, gonococcal strains with high-level azithromycin resistance (MIC ≥ 256 mg/L) appear to be resistant also to solithromycin (MICs = 4-32 mg/L) [132]. Solithromycin is well absorbed orally, with high plasma levels, intracellular concentrations and tissue distribution, has a long post-antimicrobial effect, and a 1.6 g × 1 oral dose is well-tolerated [134]. A minor Phase 2 single-center, open-label study showed that solithromycin (1.2 g × 1) treated all 22 evaluable patients with uncomplicated urogenital gonorrhoea [135]. An open-label, randomized, multi-centre Phase 3 clinical trial is currently recruiting participants with uncomplicated urogenital gonorrhoea. The study aims to include 300 participants and solithromycin 1 g × 1 orally will be compared to a dual antimicrobial regimen, i.e. ceftriaxone 500 mg × 1 plus azithromycin 1 g × 1 (www.clinicaltrials.gov).

Despite that derivates of “old” antimicrobials are developed, it is essential to develop novel antimicrobial targets, compounds and treatment strategies. Drugs with multiple targets might be crucial to mitigate resistance emergence. Recent years, several antimicrobials or other compounds, using new targets or antibacterial strategies, have been developed and shown a potent *in vitro* activity against gonococcal isolates. E.g., new protein synthesis inhibitors such as pleuromutilin BC-3781 and the boron-containing inhibitor AN3365; LpxC inhibitors; species-specific FabI inhibitors such as MUT056399; and novel bacterial topoisomerase inhibitors with target(s) different from the fluoroquinolones such as VXc-486 (also known as VT12-008911) and ETX0914 (also known as AZD0914) [136–143]. The novel oral spiropyrimidinetrione ETX0914, which additionally has a new mode-of-action [144, 145], is most advanced in the development. No resistance was initially observed examining 250 temporally, geographically and genetically diverse isolates including many fluoroquinolone-, ESC- and multidrug-resistant isolates [141]. Recently, it was shown that the susceptibility to ETX0914 among 873 contemporary clinical isolates from 21 European countries was high and no resistance was indicated [143]. ETX0914 administered orally has good target tissue penetrance, good bioavailability, high safety and tolerability (200–4000 mg × 1 orally well tolerated in healthy adult subjects in both fed and fasted state) as indicated from initial animal toxicology study and Phase 1, randomized, placebo-controlled trial conducted in 48 healthy subjects [146, 147]. An open-label, randomized, multi-centre Phase 2 clinical trial is currently recruiting patients with uncomplicated urogenital gonorrhoea. The study aims to include 180 participants and treatment with ETX0914 2 g orally (n = 70) and ETX0914 3 g orally (n = 70) will be evaluated against ceftriaxone 500 mg (n = 40) (www.clinicaltrials.gov).

## Conclusions

Dual antimicrobial therapy of gonorrhoea (ceftriaxone 250 mg-1 g plus azithromycin 1–2 g [61–66]) appears currently effective and should be considered in all settings where local quality assured AMR data do not support other therapeutic options. These dual antimicrobial regimens may not entirely prevent resistance emergence in gonococci [3, 8, 78], but they will mitigate the spread of resistant strains. Unfortunately, the first failure with dual antimicrobial therapy will most likely soon be verified. Novel affordable antimicrobials for monotherapy or at least inclusion in new dual treatment regimens for gonorrhoea are essential and several of the recently developed antimicrobials deserve increased attention. *In vitro* activity studies examining collections of geographically, temporally and genetically diverse gonococcal isolates, including MDR strains, particularly with ESC resistance and azithromycin resistance are important. Furthermore, knowledge regarding effects and biological fitness of current and emerging (*in vitro* selected and *in vivo* emerged) genetic resistance mechanisms for these antimicrobials, prediction of resistance emergence, time-kill curve analysis to evaluate antibacterial activity, and correlates between genetic and phenotypic laboratory parameters, and clinical treatment outcomes, would also be valuable. Subsequently, appropriately designed, randomized and controlled clinical trials evaluating efficacy, ideal dose, adverse effects, cost, and pharmacokinetic/pharmacodynamics data for anogenital and, importantly, also pharyngeal gonorrhoea, i.e. because treatment failures initially emerge at this anatomical site, are crucial. Finally, several examples of “thinking out of the box” for future management of gonorrhoea have also been developed recently [3] and now is certainly the right time to readdress the challenges of developing a gonococcal vaccine [148].

## Abbreviations

WHO: World Health Organization
AMR: Antimicrobial resistance
IM: Intramuscularly
IV: Intravenously
MIC: Minimum inhibitory concentration
*f*T_>MIC_: Simulation of time of free ceftriaxone above MIC
MLST: Multilocus sequence typing
NG-MAST: *N. gonorrhoeae* multi-antigen sequence typing
ND: Not determined
ST: Sequence type
XDR: Extensively drug-resistant
MSM: Men-who-have-sex-with-men
PBP2: Penicillin-binding protein 2
NAAT: Nucleic acid amplification test
ECDC: European Centre for Disease Prevention and Control
CDC: Centers for Disease Control and Prevention
POC: Point of care
CI: Confidence interval
MDR: Multidrug resistance
STI: Sexually transmitted infection
